# Supplementary material for: An observational cohort study comparing ibuprofen and oxycodone in children with fractures
Source: PLoS One. 2021 Sep 9;16(9):e0257021. doi: 10.1371/journal.pone.0257021 (PMC8428788; doi:10.1371/journal.pone.0257021)
Supplement: S1 File — (PDF) [file pone.0257021.s001.pdf]

women & children's
health research institute

Data Collection Form

The Genomics Study

Dr. S. Ali

Protocol No:	SA-TGS-085
Form Version:	29 Sep 2011

Subject ID: _ _ _ _

Subject Initials: _ _ _

Study Arm:
- O Ibuprofen
- O Codeine
- O Oxycodone

Hospital Label	DNA Tube Label

Alberta Health Services

UNIVERSITY OF ALBERTA

Protocol Number: SA-TGS-085

Short Title: The Genomics Study

Principal Investigator: Dr. Samina Ali
Department of Pediatrics
University of Alberta
11402-University Ave.
Edmonton, Alberta, Canada T6G 2J3

Study Team: Dr. David Johnson (Calgary),
Dr. Amy Drendel (Wisconsin),
Dr. Rhonda Rosychuk (Edmonton),
Dr. Patrick McGrath (Halifax)

Grant Support/Funding: Canadian Institutes of Health Research
Grant Number DSA 103534

Study Product: N/A

Protocol: SA-TGS-085<br>*The Genomics Study*	Subject ID	Subject Initials	Event Date
Event: **ED Visit**	_ _ _ _ _ _	_ _ _	_ _ / _ _ _ / 2 0 _ _<br>d d / m o n / y y y y

Subjects may be screened prior to consent, however this data cannot be recorded in the study database unless the subject has consented.

Inclusion Criteria

1. Does this patient have a fracture?	○ Yes<br>○ No
2. Is this child between 4-16 years old?	○ Yes<br>○ No
3. Is this child going to be discharged home?	○ Yes<br>○ No
4. Is this child being sent home with a recommendation to use IBUPROFEN, CODEINE or OXYCODONE?	○ Yes<br>○ No

IF YOU ANSWER 'YES' TO **ALL** OF THE ABOVE, PLEASE PROCEED.

Exclusion Criteria

1. Is this child currently taking chronic pain medications?	○ Yes<br>○ No
2. Has this child been prescribed any one of the study drugs (ibuprofen, codeine, oxycodone) at the same time?	○ Yes<br>○ No
3. Is this child cognitively unable to **self-report** pain?	○ Yes<br>○ No
4. Is the family unable to communicate in English?	○ Yes<br>○ No
5. Does the family **lack** regular (daily) telephone access?	○ Yes<br>○ No
6. Is injury greater than 24 hours old?	○ Yes<br>○ No

IF YOU ANSWERED 'YES' TO **ANY** OF THE ABOVE, PLEASE STOP NOW.

Protocol: SA-TGS-085<br>*The Genomics Study*	Subject ID	Subject Initials
Event: **ED Visit**	__ __ __ __ __	__ __ __

Informed Consent

1. Has written informed consent been obtained?<br>*If informed consent has not been obtained study assessments cannot be performed and no information can be recorded for this subject*	○ Yes<br>○ No
2. Has a copy of signed informed consent been given to the parent/guardian?	○ Yes<br>○ No
3. Date of Informed Consent/Assent:	__ __ / __ __ __ / 2 0 __ __
4. Has written assent been obtained?	○ Yes<br>○ No
a. If no, document the reason: ________________________	
b. If yes, has a copy of the signed assent been given to the subject?	○ Yes<br>○ No

Concurrent Studies

5. Is the subject currently participating in any other study in the ED?	○ Yes<br>○ No
a. If yes, please indicate which study:<br>________________________<br>________________________	

ED Visit Information

1. Date of Visit	__ __ / __ __ __ / 2 0 __ __
2. Time of Presentation	__ __ : __ __ (24 hour clock)
3. Time of Injury	__ __ : __ __ (24 hour clock)

Protocol: SA-TGS-085<br>*The Genomics Study*	Subject ID	Subject Initials
Event: **ED Visit**	_ _ _ _ _	_ _ _

Demographics

Participants date of birth and gender, located on the ID label on the cover of this form, should be entered into the OpenClinica subject record.

1. Weight (include decimals if known):	[______.___] *kg*
2. What race or ethnicity do you identify your child as?: **(must ask caregiver, directly)**	☐ Caucasian ☐ Aboriginal<br>☐ Métis ☐ Black<br>☐ Asian ☐ Arab<br>☐ East Indian ☐ Hispanic<br>☐ Other (Specify)<br>________________<br>☐ Declined to Answer

Prior Medications

1. Did the patient take any pain medications within the 24 hours before arrival in the ED?	○ Yes (If so record below)<br>○ No

Name<br>(e.g., Cloxacillin)	Dose<br>(e.g., 1-2 g)	Route		
		PO	IV	Other

Protocol: SA-TGS-085<br>*The Genomics Study*	Subject ID	Subject Initials
Event: **ED Visit**	_ _ _ _ _	_ _ _

Treatments ordered in the Emergency Department

1. Were any medications ordered in the emergency department?	○ Yes (If so record below)<br>○ No

2. Please list all medications ordered in the emergency department.				
Name<br>(e.g., Ibuprofen)	Dose<br>(e.g. 200mg)	Route		
		PO	IV	Other

3. Did this patient require procedural sedation? (meds will be recorded in the table above)	○ Yes<br>○ No
4. Was the fracture reduced in ED?	○ Yes<br>○ No

5. Were any other treatments administered in the ED (i.e. splint, sling, ice, cast)? If so please enter each treatment on a separate line below.	○ Yes<br>○ No

Protocol: SA-TGS-085<br>*The Genomics Study*	Subject ID	Subject Initials
Event: **ED Visit**	_ _ _ _ _	_ _ _

Suggested Medications for Home Use

1. Were any medications suggested or prescribed at discharge?	○ Yes (If so record below)<br>○ No

2. Please list all medications suggested or prescribed at discharge.

Name (e.g., Ibuprofen)	Dose (e.g. 200mg, if known)	Suggested frequency of use (if known)	Route	
			PO	Other

3. Did the patient receive a written prescription for medication?	○ Yes<br>○ No
4. If YES, then please transcribe the prescription below:	

5. Other treatments suggested for pain (i.e. splint, sling, ice, cast)? If so please enter each treatment on a separate line below.	○ Yes<br>○ No

Protocol: SA-TGS-085<br>*The Genomics Study*	Subject ID	Subject Initials
Event: **ED Visit**	_ _ _ _ _ _	_ _ _

ED Visit Outcome

1. Date of discharge from the ED?	_ _ / _ _ _ / 2 0 _ _
2. Time of discharge	_ _ : _ _ (24 hour clock)
3. What was the patient discharge plan?<br>(Tick all that apply.)	○ Not charted/ not able to ask MD/RN<br>○ Return to ED, prn (anytime)<br>○ Return to ED, scheduled<br>○ Follow-up with family MD<br>○ Referral to Orthopedic Surgery for follow-up<br>○ Other:<br>______________________
4. Discharge diagnosis:	______________________

Study Arm

The study arm is decided by the medication prescribed or suggested for use at home and is entered into the subject record of OpenClinica. Please document the study arm on the front cover of this data collection booklet.

Coordinator Signature

Signed:	______________________	Date:	_ _ / _ _ _ / 2 0 _ _

Protocol: SA-TGS-085<br>*The Genomics Study*	Subject ID	Subject Initials	Event Date
Event: **Follow-up Day 1**	_ _ _ _ _ _	_ _ _	_ _ / _ _ _ / 2 0 _ _<br>d d / mon / y y y y

Contact Information

1. Date call due:	_ _ / _ _ _ / 2 0 _ _

2. Attempted Contacts

Date	Time (24 hour clock)	RA / Nurse Initials

3. Who did you speak to on the phone?<br>(Check as many as apply)	○ Parent<br>○ Child<br>○ Primary Caregiver (other than parent)<br>○ Other<br>________________________________

Protocol: SA-TGS-085<br>*The Genomics Study*	Subject ID	Subject Initials
Event: **Follow-up Day 1**	_ _ _ _ _	_ _ _

Pain Scores and Treatment

1. Were there any complaints of pain in the last 24 hours?<br>If **yes**, please complete the questions below.	○ Yes<br>○ No
a. What was your/ your child's SELF-REPORTED **average** pain score today (0-10 as per FPS-R)?	___
b. What was your/ your child's SELF-REPORTED **minimum** pain score today (0-10 as per FPS-R)?	___
c. What was your/ your child's SELF-REPORTED **maximum** pain score today (0-10) as per FPS-R)?	___
d. Did you treat that maximum pain with medication/medicine?	○ Yes<br>○ No
e. If you treated the pain with medication what was your/your child's SELF-REPORTED pain score 1 hour after the medication/medicine (0-10)?	___

Medication Used to Treat Maximum Pain

**Indication**	**Medication used?** Y/N	**Name of Medication used**	**Date** (dd/mon/yyyy)	**Time** (24 hr clock)	**Dose** (include units)
For **Maximum** Pain in the last 24 hours.					

Protocol: SA-TGS-085<br>*The Genomics Study*	Subject ID	Subject Initials
Event: **Follow-up Day 1**	_ _ _ _ _	_ _ _

Medications Used to Treat Pain (other than maximum pain)

All answers pertain to the last 24 hours.

**Indication**	**Medication used?** Y/N	**Name of Medication used**	**Date** (dd/mon/yyyy)	**Time** (24 hr clock)	**Dose** (include units)
Codeine in the last 24 hours/?					
Acetaminophen in the last 24 hours?					
Ibuprofen in the last 24 hours?					
Oxycodone in the last 24 hours?					
Other pain medications in the last 24 hours?					

Non-Pharmacologic Pain Treatments

1. Are you (is your child) using any other treatments for pain (i.e. splint, sling, ice, cast)?<br>If yes, please enter each treatment on a separate line below.	○ Yes<br>○ No

Protocol: SA-TGS-085<br>*The Genomics Study*	Subject ID	Subject Initials
Event: **Follow-up Day 1**	_ _ _ _ _	_ _ _

Limitation of Activities

"We know that having a broken bone can limit activities, but we are particularly interested in whether the PAIN affected your child's activities."

1. Did **fracture pain** affect you/your child's ability to **eat** normally today?	○ Yes<br>○ No
2. Did **fracture pain** affect you/your child's ability to **sleep** normally today?	○ Yes<br>○ No
3. Did **fracture pain** affect you/your child's ability to **go to school** normally today ?	○ Yes<br>○ No
4. Did **fracture pain** affect you/your child's ability to **play** normally today?	○ Yes<br>○ No

Medical Follow-up

1. Day 1 -Have you / your child seen a doctor since the original emergency department visit?<br>Or:<br>Day 2 or 3 - Have you / your child seen a doctor since the last follow-up call?	○ Yes<br>○ No
2. If yes, please specify:	○ ER physician,<br>○ Pediatrician<br>○ Family Doctor<br>○ Specialist:
What was the reason for the visit to the doctor?	
______________________________<br>______________________________	

Protocol: SA-TGS-085<br>*The Genomics Study*	Subject ID	Subject Initials
Event: **Follow-up Day 1**	_ _ _ _ _	_ _ _

Adverse Events

**On a scale of 0-10**, what number describes the extent of *<insert symptom name, here>* that you/ your child has experienced today? Zero means none and ten means the worst ever.	
Nausea	[ ]
Vomiting	[ ]
Abdominal pain	[ ]
Drowsiness	[ ]
Dizziness	[ ]
Constipation	[ ]
Lack of appetite	[ ]
Rash	[ ]
Other (Please specify below)	[ ]

Serious Adverse Events

1. Have you / your child suffered any serious side effects such as death, anaphylaxis, or any event requiring hospitalization?	○ Yes<br>○ No
2. If yes please complete the details below:	
Serious Adverse Event Details	Outcome (i.e. admitted, ED visit)

Coordinator Signature

Signed:	______________________	Date:	_ _ / _ _ _ / 2 0 _ _

Please thank the family for their participation.

Protocol: SA-TGS-085<br>*The Genomics Study*	Subject ID	Subject Initials	Event Date
Event: **Follow-up Day 2**	_ _ _ _ _ _	_ _ _	_ _ / _ _ _ / 2 0 _ _<br>d d / m o n / y y y y

Contact Information

1. Date call due:	_ _ / _ _ _ / 2 0 _ _

2. Attempted Contacts		
Date	Time (24 hour clock)	RA / Nurse Initials

3. Who did you speak to on the phone?<br>(Check as many as apply)	○ Parent<br>○ Child<br>○ Primary Caregiver (other than parent)<br>○ Other<br>________________________________

Protocol: SA-TGS-085<br>*The Genomics Study*	Subject ID	Subject Initials
Event: **Follow-up Day 2**	_ _ _ _ _	_ _ _

Pain Scores and Treatment

1. Were there any complaints of pain in the last 24 hours?<br>If **yes**, please complete the questions below.	○ Yes<br>○ No
a. What was your/ your child's SELF-REPORTED **average** pain score today (0-10 as per FPS-R)?	___
b. What was your/ your child's SELF-REPORTED **minimum** pain score today (0-10 as per FPS-R)?	___
c. What was your/ your child's SELF-REPORTED **maximum** pain score today (0-10) as per FPS-R)?	___
d. Did you treat that maximum pain with medication/medicine?	○ Yes<br>○ No
e. If you treated the pain with medication what was your/your child's SELF-REPORTED pain score 1 hour after the medication/medicine (0-10)?	___

Medication Used to Treat Maximum Pain

**Indication**	**Medication used?** Y/N	**Name of Medication used**	**Date** (dd/mon/yyyy)	**Time** (24 hr clock)	**Dose** (include units)
For **Maximum** Pain in the last 24 hours.					

Protocol: SA-TGS-085<br>*The Genomics Study*	Subject ID	Subject Initials
Event: **Follow-up Day 2**	_ _ _ _ _	_ _ _

Medications Used to Treat Pain (other than maximum pain)

All answers pertain to the last 24 hours.

**Indication**	**Medication used?** Y/N	**Name of Medication used**	**Date** (dd/mon/yyyy)	**Time** (24 hr clock)	**Dose** (include units)
Codeine in the last 24 hours/?					
Acetaminophen in the last 24 hours?					
Ibuprofen in the last 24 hours?					
Oxycodone in the last 24 hours?					
Other pain medications in the last 24 hours?					

Non-Pharmacologic Pain Treatments

1. Are you (is your child) using any other treatments for pain (i.e. splint, sling, ice, cast)?<br>If yes, please enter each treatment on a separate line below.	○ Yes<br>○ No

Protocol: SA-TGS-085<br>*The Genomics Study*	Subject ID	Subject Initials
Event: **Follow-up Day 2**	_ _ _ _ _	_ _ _

Limitation of Activities

"We know that having a broken bone can limit activities, but we are particularly interested in whether the PAIN affected your child's activities."

1. Did **fracture pain** affect you/your child's ability to **eat** normally today?	○ Yes<br>○ No
2. Did **fracture pain** affect you/your child's ability to **sleep** normally today?	○ Yes<br>○ No
3. Did **fracture pain** affect you/your child's ability to **go to school** normally today ?	○ Yes<br>○ No
4. Did **fracture pain** affect you/your child's ability to **play** normally today?	○ Yes<br>○ No

Medical Follow-up

1. Day 1 -Have you / your child seen a doctor since the original emergency department visit?<br>Or:<br>Day 2 or 3 - Have you / your child seen a doctor since the last follow-up call?	○ Yes<br>○ No
2. If yes, please specify:	○ ER physician,<br>○ Pediatrician<br>○ Family Doctor<br>○ Specialist:
What was the reason for the visit to the doctor?	
______________________________<br>______________________________	

Protocol: SA-TGS-085<br>*The Genomics Study*	Subject ID	Subject Initials
Event: **Follow-up Day 2**	_ _ _ _ _	_ _ _

Adverse Events

**On a scale of 0-10**, what number describes the extent of *<insert symptom name, here>* that you/ your child has experienced today? Zero means none and ten means the worst ever.	
Nausea	[ ]
Vomiting	[ ]
Abdominal pain	[ ]
Drowsiness	[ ]
Dizziness	[ ]
Constipation	[ ]
Lack of appetite	[ ]
Rash	[ ]
Other (Please specify below)	[ ]

Serious Adverse Events

1. Have you / your child suffered any serious side effects such as death, anaphylaxis, or any event requiring hospitalization?	○ Yes<br>○ No
2. If yes please complete the details below:	
Serious Adverse Event Details	Outcome (i.e. admitted, ED visit)

Coordinator Signature

Signed:	______________________________	Date:	_ _ / _ _ _ / 2 0 _ _

Please thank the family for their participation.

Protocol: SA-TGS-085<br>*The Genomics Study*	Subject ID	Subject Initials	Event Date
Event: **Follow-up Day 3**	_ _ _ _ _ _	_ _ _	_ _ / _ _ _ / 2 0 _ _<br>dd / mon / yyyy

Contact Information

1. Date call due:	_ _ / _ _ _ / 2 0 _ _

2. Attempted Contacts		
Date	Time (24 hour clock)	RA / Nurse Initials

3. Who did you speak to on the phone?<br>(Check as many as apply)	○ Parent<br>○ Child<br>○ Primary Caregiver (other than parent)<br>○ Other<br>______________________________

Protocol: SA-TGS-085<br>*The Genomics Study*	Subject ID	Subject Initials
Event: **Follow-up Day 3**	_ _ _ _ _	_ _ _

Pain Scores and Treatment

1. Were there any complaints of pain in the last 24 hours?<br>If **yes**, please complete the questions below.	○ Yes<br>○ No
a. What was your/ your child's SELF-REPORTED **average** pain score today (0-10 as per FPS-R)?	___
b. What was your/ your child's SELF-REPORTED **minimum** pain score today (0-10 as per FPS-R)?	___
c. What was your/ your child's SELF-REPORTED **maximum** pain score today (0-10) as per FPS-R)?	___
d. Did you treat that maximum pain with medication/medicine?	○ Yes<br>○ No
e. If you treated the pain with medication what was your/your child's SELF-REPORTED pain score 1 hour after the medication/medicine (0-10)?	___

Medication Used to Treat Maximum Pain

**Indication**	**Medication used?** Y/N	**Name of Medication used**	**Date** (dd/mon/yyyy)	**Time** (24 hr clock)	**Dose** (include units)
For **Maximum** Pain in the last 24 hours.					

Protocol: SA-TGS-085<br>*The Genomics Study*	Subject ID	Subject Initials
Event: **Follow-up Day 3**	_ _ _ _ _	_ _ _

Medications Used to Treat Pain (other than maximum pain)

All answers pertain to the last 24 hours.

**Indication**	**Medication used?** Y/N	**Name of Medication used**	**Date** (dd/mon/yyyy)	**Time** (24 hr clock)	**Dose** (include units)
Codeine in the last 24 hours/?					
Acetaminophen in the last 24 hours?					
Ibuprofen in the last 24 hours?					
Oxycodone in the last 24 hours?					
Other pain medications in the last 24 hours?					

Non-Pharmacologic Pain Treatments

1. Are you (is your child) using any other treatments for pain (i.e. splint, sling, ice, cast)?<br>If yes, please enter each treatment on a separate line below.	○ Yes<br>○ No

Protocol: SA-TGS-085<br>*The Genomics Study*	Subject ID	Subject Initials
Event: **Follow-up Day 3**	_ _ _ _ _	_ _ _

Limitation of Activities

"We know that having a broken bone can limit activities, but we are particularly interested in whether the PAIN affected your child's activities."

1. Did **fracture pain** affect you/your child's ability to **eat** normally today?	○ Yes<br>○ No
2. Did **fracture pain** affect you/your child's ability to **sleep** normally today?	○ Yes<br>○ No
3. Did **fracture pain** affect you/your child's ability to **go to school** normally today ?	○ Yes<br>○ No
4. Did **fracture pain** affect you/your child's ability to **play** normally today?	○ Yes<br>○ No

Medical Follow-up

1. Day 1 -Have you / your child seen a doctor since the original emergency department visit?<br>Or:<br>Day 2 or 3 - Have you / your child seen a doctor since the last follow-up call?	○ Yes<br>○ No
2. If yes, please specify:	○ ER physician,<br>○ Pediatrician<br>○ Family Doctor<br>○ Specialist:
What was the reason for the visit to the doctor?	
____<br>____	

Protocol: SA-TGS-085<br>*The Genomics Study*	Subject ID	Subject Initials
Event: **Follow-up Day 3**	_ _ _ _ _	_ _ _

Adverse Events

**On a scale of 0-10**, what number describes the extent of *<insert symptom name, here>* that you/ your child has experienced today? Zero means none and ten means the worst ever.	
Nausea	[ ]
Vomiting	[ ]
Abdominal pain	[ ]
Drowsiness	[ ]
Dizziness	[ ]
Constipation	[ ]
Lack of appetite	[ ]
Rash	[ ]
Other (Please specify below)	[ ]

Serious Adverse Events

1. Have you / your child suffered any serious side effects such as death, anaphylaxis, or any event requiring hospitalization?	○ Yes<br>○ No
2. If yes please complete the details below:	
Serious Adverse Event Details	Outcome (i.e. admitted, ED visit)

Coordinator Signature

Signed:	______________________________	Date:	_ _ / _ _ _ / 2 0 _ _

Please thank the family for their participation.

Protocol: SA-TGS-085<br>*The Genomics Study*	Subject ID	Subject Initials	Event Date
Event: **Follow-up Week 2**	_ _ _ _ _	_ _ _	_ _ / _ _ _ / 2 0 _ _<br>d d / m o n / y y y y

Contact Information

1. Date call due:	_ _ / _ _ _ / 2 0 _ _

2. Attempted Contacts		
Date	Time (24 hour clock)	RA / Nurse Initials

3. Who did you speak to on the phone?<br>(Check as many as apply)	○ Parent<br>○ Child<br>○ Primary Caregiver (other than parent)<br>○ Other<br>________________________________

Protocol: SA-TGS-085<br>*The Genomics Study*	Subject ID	Subject Initials
Event: **Follow-up Week 2**	__ __ __ __ __	__ __ __

Pain Scores

1. Have you/ has your child complained of fracture-related pain in the last 48 hours?	○ Yes<br>○ No
2. If **yes**, what is your/ your child's SELF-REPORTED **average** pain score over the last 48 hours (0-10 as per FPS-R chart)?	___
3. Any comments:	

Medications and Treatments

1. Have you/ has your child used **pain medicine** for the fracture in the last 48 hours?<br>If **yes,** please enter the name of each medication on a separate line below.	○ Yes<br>○ No

2. Have you/ has your child used any other treatments **for pain** (i.e. splint, sling, ice, cast) in the last 48 hours?<br>If **yes**, please enter each treatment on a separate line below.	○ Yes<br>○ No

Protocol: SA-TGS-085<br>*The Genomics Study*	Subject ID	Subject Initials
Event: **Follow-up Week 2**	_ _ _ _ _	_ _ _

Limitation of Activities

"We know that having a broken bone can limit activities, but we are particularly interested in whether the PAIN affected your child's activities."

1. Did **fracture pain** you/your child's ability to **eat** normally in the last 48 hours?	○ Yes<br>○ No
2. Did **fracture pain** you/your child's ability to **sleep** normally in the last 48 hours?	○ Yes<br>○ No
3. Did **fracture pain** you/your child's ability to **go to school** normally in the last 48 hours?	○ Yes<br>○ No
4. Did **fracture pain** you/your child's ability to **play** normally in the last 48 hours?	○ Yes<br>○ No

Protocol: SA-TGS-085<br>*The Genomics Study*	Subject ID	Subject Initials
Event: **Follow-up Week 2**	_ _ _ _ _	_ _ _

Diagnosis and Follow-up

1. Have you / your child seen a doctor since the last follow-up call?	○ Yes<br>○ No
2. If no why not?	
____	
3. If yes, please specify:	○ ER physician,<br>○ Pediatrician<br>○ Family Doctor<br>○ Specialist:
a. What did they say at follow-up?	
____<br>____	
b. Did your/your child's diagnosis change since your first ED visit?	○ Yes<br>○ No
c. If **yes** then what is the new diagnosis?	
____	
d. Did you / your child have to go to the operating room or have an operation?	○ Yes<br>○ No
e. Did your doctor ever say they were worried about the broken bone not healing well?	○ Yes<br>○ No
If yes, did they explain why they were worried, and what it might be due to?	
____	

Coordinator Signature

Signed:	______________________________	Date:	_ _ / _ _ _ / 2 0 _ _

Please thank the family for their participation.

Protocol: SA-TGS-085<br>*The Genomics Study*	Subject ID	Subject Initials	Event Date
Event: **Follow-up Week 6**	_ _ _ _ _ _	_ _ _	_ _ / _ _ _ / 2 0 _ _<br>d d / m o n / y y y y

Contact Information

1. Date call due:	_ _ / _ _ _ / 2 0 _ _

2. Attempted Contacts		
Date	Time (24 hour clock)	RA / Nurse Initials

3. Who did you speak to on the phone?<br>(Check as many as apply)	○ Parent<br>○ Child<br>○ Primary Caregiver (other than parent)<br>○ Other<br>______________________________

Protocol: SA-TGS-085<br>*The Genomics Study*	Subject ID	Subject Initials
Event: **Follow-up Week 6**	_ _ _ _ _	_ _ _

Pain Scores

1. Have you/ has your child complained of fracture-related pain in the last 48 hours?	○ Yes<br>○ No
2. If **yes**, what is your/ your child's SELF-REPORTED **average** pain score over the last 48 hours (0-10 as per FPS-R chart)?	___
3. Any comments:	
______________________________<br>______________________________	

Medications and Treatments

1. Have you/ has your child used **pain medicine** for the fracture in the last 48 hours?<br>If **yes,** please enter the name of each medication on a separate line below.	○ Yes<br>○ No

2. Have you/ has your child used any other treatments **for pain** (i.e. splint, sling, ice, cast) in the last 48 hours?<br>If **yes**, please enter each treatment on a separate line below.	○ Yes<br>○ No

Protocol: SA-TGS-085<br>*The Genomics Study*	Subject ID	Subject Initials
Event: **Follow-up Week 6**	_ _ _ _ _	_ _ _

Limitation of Activities

"We know that having a broken bone can limit activities, but we are particularly interested in whether the PAIN affected your child's activities."

1. Did **fracture pain** you/your child's ability to **eat** normally in the last 48 hours?	○ Yes<br>○ No
2. Did **fracture pain** you/your child's ability to **sleep** normally in the last 48 hours?	○ Yes<br>○ No
3. Did **fracture pain** you/your child's ability to **go to school** normally in the last 48 hours?	○ Yes<br>○ No
4. Did **fracture pain** you/your child's ability to **play** normally in the last 48 hours?	○ Yes<br>○ No

Protocol: SA-TGS-085<br>*The Genomics Study*	Subject ID	Subject Initials
Event: **Follow-up Week 6**	_ _ _ _ _	_ _ _

Diagnosis and Follow-up

1. Have you / your child seen a doctor since the last follow-up call?	○ Yes<br>○ No
2. If no why not?	
______________________________	
3. If yes, please specify:	○ ER physician,<br>○ Pediatrician<br>○ Family Doctor<br>○ Specialist:
a. What did they say at follow-up?	
______________________________<br>______________________________	
b. Did your/your child's diagnosis change since your first ED visit?	○ Yes<br>○ No
c. If **yes** then what is the new diagnosis?	
______________________________	
d. Did you / your child have to go to the operating room or have an operation?	○ Yes<br>○ No
e. Did your doctor ever say they were worried about the broken bone not healing well?	○ Yes<br>○ No
If yes, did they explain why they were worried, and what it might be due to?	
______________________________	

Our thank-you

If you wish to receive a 5$ Tim Horton’s coffee card or Toys'R'Us gift certificate as a token of our thanks for completing this study, please provide us with your mailing address:

Coordinator Signature

Signed:	______________________________	Date:	_ _ / _ _ _ / 2 0 _ _

Please thank the family for their participation.

Protocol: SA-TGS-085 *The Genomics Study*	Subject ID	Subject Initials	Event Date
Event: **End of Study**	_ _ _ _ _	_ _ _	_ _ / _ _ _ / 2 0 _ _<br>d d / mon / y y y y

Study Completion

1. Did the patient complete the study?	○ Yes<br>○ No
a. If not, why not?	○ Lost to follow-up<br>○ Withdrew consent<br>○ Adverse event<br>○ Non-compliance<br>○ Other (please comment)
b. Comment:	______________________<br>______________________<br>______________________<br>______________________
2. Date of discontinuation or completion.	_ _ / _ _ _ / 2 0 _ _
3. Was the DNA sample successfully analyzed?	○ Yes<br>○ No
a. If not, why not?	______________________<br>______________________<br>______________________

Coordinator Signature

Signed:	______________________________	Date:	_ _ / _ _ _ / 2 0 _ _
