# Supplementary material for: An observational cohort study comparing ibuprofen and oxycodone in children with fractures
Source: PLoS One. 2021 Sep 9;16(9):e0257021. doi: 10.1371/journal.pone.0257021 (PMC8428788; doi:10.1371/journal.pone.0257021)
Supplement: Attachment — Submitted filename: Response to the Editors.docx [file pone.0257021.s003.docx]

To:

PLOS ONE Staff Editors

MS ID#: PONE-D-20-33147

Dear Editors:

Thank you very much for asking me to review the paper entitled *"* An observational cohort study comparing ibuprofen and oxycodone in children with fractures*”* by Ali S. et al, which has been submitted for publication in PLOS ONE Journal.

Ali and coll. made a prospective observational cohort study to compare the effectiveness and safety of ibuprofen and oxycodone for at-home management of an isolated fracture pain in 329 children aged 4-16 years (n=217 ibuprofen, n=112 oxycodone). They found that ibuprofen and oxycodone have similar analgesic effectiveness for at-home fracture pain, but oxycodone was associated with more adverse events and negatively impacted function. The study is well conducted and the topic is of interest while confirmatory of the efficacy and safety of ibuprofen for fracture-related pain.

My only concern regards the presence of a selection bias in the administration of oxycodone in children with a more severe fracture. In fact, oxycodone was more often prescribed in fractures needing procedural sedation and reduction, and in fractures later referred to the orthopedic surgeon. Also, more fractures involving the upper leg/hip, suspected for being higher energy injuries, were reported in the oxycodone group, while buckle fractures, low energy trauma, were fewer. This might suggest that fractures deemed clinically more important were selected into this group. This bias could also explain the more frequent use of adjuvant medications during the first 3 days in the oxycodone group compared to the ibuprofen group and, as a consequence, the higher adverse events incidence in children treated with oxycodone.

In my opinion, this study weakness, which is part of its observational nature, should be more highlighted and discussed.

Sincerely yours,

Simone Lazzeri, MD, MSc
