# Supplementary material for: An observational cohort study comparing ibuprofen and oxycodone in children with fractures
Source: PLoS One. 2021 Sep 9;16(9):e0257021. doi: 10.1371/journal.pone.0257021 (PMC8428788; doi:10.1371/journal.pone.0257021)
Supplement: Attachment — Submitted filename: TABLE - Ali - PLOS ONE 21June2021.docx [file pone.0257021.s004.docx]

**Response to Reviewers: PLOS ONE (April 2021)**

*"An observational cohort study comparing ibuprofen and oxycodone in children with fractures"*

| **Reviewer** | **Section** | **Comment** | **Response** |
| --- | --- | --- | --- |
| Journal Require-ments |  | Please ensure that your manuscript meets PLOS ONE's style requirements, including those for file naming. | This has been reviewed and edited to journal specifications. |
|  |  | Please include additional information regarding the survey or questionnaire used in the study and ensure that you have provided sufficient details that others could replicate the analyses. For instance, if you developed a questionnaire as part of this study and it is not under a copyright more restrictive than CC-BY, please include a copy, in both the original language and English, as Supporting Information. | The study case report form, including follow up survey tools, has been included as Supporting Information File 1. |
|  |  | Please clarify the sources of funding (financial or material support) for your study.  List the grants or organizations that supported your study, including funding received from your institution.  State what role the funders took in the study. If the funders had no role in your study, please state: “The funders had no role in study design, data collection and analysis, decision to publish, or preparation of the manuscript.”  If any authors received a salary from any of your funders, please state which authors and which funders.  If you did not receive any funding for this study, please state: “The authors received no specific funding for this work.”    Please include your amended statements within your cover letter; we will change the online submission form on your behalf. | The CIHR was the sole funder and this has been acknowledged.  This is already provided and has been restated in the revision cover letter.  This has been added to the new cover letter.  Thank you. |
|  |  | We note that you have indicated that data from this study are available upon request. PLOS only allows data to be available upon request if there are legal or ethical restrictions on sharing data publicly. For information on unacceptable data access restrictions, please see http://journals.plos.org/plosone/s/data-availability#loc-unacceptable-data-access-restrictions. | Please see response that follows. We are not able to share our full dataset at this time. |
|  |  | In your revised cover letter, please address the following prompts:  a) If there are ethical or legal restrictions on sharing a de-identified data set, please explain them in detail (e.g., data contain potentially identifying or sensitive patient information) and who has imposed them (e.g., an ethics committee). Please also provide contact information for a data access committee, ethics committee, or other institutional body to which data requests may be sent.  b) If there are no restrictions, please upload the minimal anonymized data set necessary to replicate your study findings as either Supporting Information files or to a stable, public repository and provide us with the relevant URLs, DOIs, or accession numbers. Please see http://www.bmj.com/content/340/bmj.c181.long for guidelines on how to de-identify and prepare clinical data for publication. For a list of acceptable repositories, please see http://journals.plos.org/plosone/s/data-availability#loc-recommended-repositories. | We have consulted with the University of Alberta ethics board and they agree with our decision to respectfully NOT make the dataset available publicly. When these families were consented for the study, the consent and assent did not include sharing of this nature as a stated objective. Thus, sharing would be without their consent. Further, our dataset includes potentially identifying information, and sensitive information including gender, diagnosis, race/ethnicity, and specific details regarding time and date of presentation. We do not feel comfortable sharing this publicly. All requests for data may be sent to Mr. Rick Watts, Team Lead, Research Informatics, Women and Children’s Health Research Institute [rick.watts@ualberta.ca](mailto:rick.watts@ualberta.ca) OR the Corresponding Author Dr. Samina Ali sali@ualberta.ca. |
|  |  | One of the noted authors is a group or consortium [on behalf of Pediatric Emergency Research Canada]. In addition to naming the author group, please list the individual authors and affiliations within this group in the acknowledgments section of your manuscript. Please also indicate clearly a lead author for this group along with a contact email address. | This has been added to the acknowledgements section. |
| 1 | Comments | The authors report a well written paper on an interesting paper. | Many thanks for the kind comment. |
|  |  | However, the study design cannot support their conclusions. The administration of oxycodone was decided by the physician and this could lead to important bias. Moreover, the lack of blindness is a further important potential bias. An RCT would be the appropriate study design for the purposes of the authors' study. | We wholly agree that an RCT would be an excellent way to test the relative efficacy and safety of oral opioids and ibuprofen. In fact, our team, building on this work and other trials that we have performed, is currently conducting a multi-centre trial of acetaminophen, ibuprofen, and oral hydromorphone (BMJ Open. 2020 Jun 21;10(6):e035177. doi: 10.1136/bmjopen-2019-035177. PMID: 32565458).  This submitted study aimed to determine the effectiveness of oxycodone and ibuprofen in the post-market, real-world, less controlled setting of at-home use. In fact, the national funding call that supported this study was specifically targeted to post-market, real world evaluation of drugs. This observational study allowed us to comment on the effectiveness of oxycodone and ibuprofen, as it would naturally be used. This was not an efficacy trial. We wanted to study *how families used the medication when they were able to make the decisions for use on their own*, and without researcher-given instructions. Thus, a more pragmatic and observational approach was employed.  We have added the following to our Discussion section to reflect these concerns.  *“It is important to note that when one considers that there may be some inherent clinical or demographic differences in the participants included in each of the cohorts, coupled with the use of other pain medications besides their original assignment, our study results do not necessarily reflect the ‘pure’ clinical efficacy of oxycodone, but rather, the clinical effectiveness of prescribing it for fracture-related pain.”* |
|  |  | However, if the authors want to consider data from an observational study design, a propensity score analysis should be performed. | Thank you for this suggestion. We acknowledge that there are strengths to employing a propensity score analysis. Unfortunately, that was not our planned analysis during the study design phase, and we were not focused on collecting data on all key factors that may be related to treatment decision and outcome. We have not measured all potential confounders. As such, a propensity score analysis in this situation may not lead to the appropriate estimates. We have focused our analyses on summary statistics and basic tests.  Further, to our understanding, an important condition for using propensity scores is that every subject has a nonzero probability to receive either treatment. We cannot say with certainty that this would have been the case for our study subjects, with our particular observational cohort design.  Your comments have definitely given us an opportunity to think about our wording in the manuscript and we have made some edits to reflect these considerations, particularly in our presentation of the results and our limitations. |
| 2 | Comments | Ali and coll. made a prospective observational cohort study to compare the effectiveness and safety of ibuprofen and oxycodone for at-home management of an isolated fracture pain in 329 children aged 4-16 years (n=217 ibuprofen, n=112 oxycodone). They found that ibuprofen and oxycodone have similar analgesic effectiveness for at-home fracture pain, but oxycodone was associated with more adverse events and negatively impacted function. The study is well conducted and the topic is of interest while confirmatory of the efficacy and safety of ibuprofen for fracture-related pain. | Many thanks for these kind comments. |
|  |  | My only concern regards the presence of a selection bias in the administration of oxycodone in children with a more severe fracture. In fact, oxycodone was more often prescribed in fractures needing procedural sedation and reduction, and in fractures later referred to the orthopedic surgeon. | We agree that those requiring fracture reduction may, in theory, experience more pain at home, but this was not the case in our study population, where maximum pain scores between cohorts demonstrated very similar values (see Table 3), with Day 1 maximum pain scores being 6.0 vs 6.3, a difference which is less than the minimal clinically important difference of 1-2.  Further, the difference between the proportion of children requiring fracture reduction/sedation was 6-7% in a 300+ patient sample which would not result in a statistically significant difference (although we do not report this as good reporting practice suggests that demographic tables should allow readers to reflect and draw conclusions, themselves, just as you have). Orthopedic follow up rates naturally follow reduction rates, as all children requiring a reduction are followed by orthopedics at our site.  We have reflected on this concern and have added the following to the limitations section:  *“Fourth, there were small demographic imbalances between the two cohorts (i.e., slightly older, more fracture reductions,* ***less buckle fractures****, and more procedural sedations in the oxycodone group).* ***This might suggest that fractures deemed clinically more important were selected into the oxycodone group****. Still, this represents the reality of who received oxycodone in our clinical setting, as opioids tend to be prescribed less for younger children, and more often after reductions.* ***These demographic imbalances should be considered when interpreting our results, in terms of applicability to local clinical populations.”***  We have also added the following to the Discussion section, along with other minor editing throughout the manuscript to reflect that the groups represented *prescribing recommendations*, and not pure clinical efficacy:  *“It is important to note that when one considers that there may be some inherent clinical or demographic differences in the participants included in each of the cohorts, coupled with the use of other pain medications besides their original assignment, our study results do not necessarily reflect the ‘pure’ clinical efficacy of oxycodone, but rather, the clinical effectiveness of prescribing it for fracture-related pain.”* |
|  |  | Also, more fractures involving the upper leg/hip, suspected for being higher energy injuries, were reported in the oxycodone group, while buckle fractures, low energy trauma, were fewer. This might suggest that fractures deemed clinically more important were selected into this group. This bias could also explain the more frequent use of adjuvant medications during the first 3 days in the oxycodone group compared to the ibuprofen group and, as a consequence, the higher adverse events incidence in children treated with oxycodone.  In my opinion, this study weakness, which is part of its observational nature, should be more highlighted and discussed. | We agree that lower limb fractures may reflect higher energy injuries. Interestingly, lower limb fractures total 20% of fractures in the ibuprofen group, and a lower 18% in the oxycodone group, in our study. Upper leg/hip fractures were indeed more common in the oxycodone group (3.6 vs 0.6%) but represent only 2 more individual children; as such we feel this individual variable is unlikely to have strongly influenced the overall results of our study.  Buckle fractures were, indeed, represented in greater numbers in the ibuprofen group than the oxycodone group. Notably, this did not affect the overall maximum pain reported between groups, on average, for each of the 3 days (see Table 3).  While each of these noted differences between groups might be perceived as seemingly minor, we appreciate your overall concerns with these minor differences *in combination*, and the need to highlight how this might affect interpretation of our results. We have made some edits to the wording of the discussion (tracked within the paper), as well as added the following to limitations, to mitigate this.  *“**Fourth, there were small demographic imbalances between the two cohorts (i.e., slightly older, more fracture reductions,* ***less buckle fractures****, and more procedural sedations in the oxycodone group).* ***This might suggest that fractures deemed clinically more important were selected into the oxycodone group.*** *Still, this represents the reality of who received oxycodone in our clinical setting, as opioids tend to be prescribed less for younger children, and more often after reductions.* ***These demographic imbalances should be considered when interpreting our results, in terms of applicability to local clinical populations.”*** |
